# Editorial: Hereditary alpha-tryptasemia: 10 years on

**DOI:** 10.3389/falgy.2026.1980092

**Published:** 2026-10-08

**Authors:** Mark Kacar, Peter Kopac, Jonathan J. Lyons

**Affiliations:** 1 Univerzitetna Klinika za Pljucne bolezni in Alergijo Golnik, Golnik, Slovenia; 2 Laboratory of Allergic Diseases, National Institute of Allergy and Infectious Diseases, Bethesda, MD, United States

**Keywords:** anaphylaxis, food allergy, hereditary alpha-tryptasaemia, mast cell, tryptase

## Abstract

Hereditary alpha-tryptasemia (H*α*T) has emerged over the past decade as an important genetic modifier of mast cell–mediated disease and anaphylaxis. First described in 2016 by Lyons and colleagues, H*α*T is an autosomal dominant genetic trait caused by increased germline copy numbers of the *TPSAB1* gene encoding *α*-tryptase. It affects approximately 4%–6% of Western populations and is now recognized as the most common cause of elevated basal serum tryptase (BST) levels in otherwise healthy individuals. Despite its high prevalence, the clinical penetrance of H*α*T is remarkably heterogeneous. Approximately one-third of individuals carrying the trait remain asymptomatic, whereas others develop multisystem manifestations that include flushing, pruritus, gastrointestinal symptoms, and recurrent systemic reactions. While associations with connective tissue abnormalities, dysautonomia, joint hypermobility, and postural orthostatic tachycardia syndrome (POTS) were initially reported, these were later disproven. Importantly, H*α*T is best understood as a genetic trait rather than a disease entity, with clinical relevance that depends strongly on context.

Most critically, H*α*T has emerged as a clinically relevant modifier of mast cell (MC) mediator–associated symptoms and immediate hypersensitivity reactions (1, 2). Although H*α*T does not intrinsically cause allergic sensitization, accumulating evidence suggests that it can significantly amplify the frequency and severity of anaphylaxis in defined clinical settings, particularly in Hymenoptera venom allergy, food allergy, idiopathic anaphylaxis, and reactions to high-risk medications, such as antibiotics and monoclonal antibodies (2–5) In addition, emerging data suggest that H*α*T may influence mast cell homeostasis and immune responses within the gastrointestinal tract (6). At the intersection of genetics and clinical allergy, H*α*T is also increasingly recognized as being associated with clonal mast cell disorders (MCDs). Several cohorts have demonstrated a disproportionately high prevalence of H*α*T in patients with systemic mastocytosis (SM) and monoclonal mast cell activation syndromes, raising important diagnostic and prognostic questions (2, 7). Whether H*α*T merely modifies disease severity or actively contributes to mast cell pathology remains unresolved.

As research in this field expands rapidly, H*α*T is increasingly recognized not simply as a biochemical curiosity but also as a clinically meaningful genetic trait that is reshaping our understanding of mast cell biology, anaphylaxis susceptibility, and precision medicine in allergy and immunology. The articles in this Research Topic reflect this evolving view of H*α*T: as a determinant of BST levels, a modifier of anaphylaxis severity, a complicating factor in MCD diagnostics, and a possible contributor to mucosal immune dysregulation.

In our Special Issue, Puxkandl et al*.* found H*α*T in 93% of individuals with increased BST levels, confirming that H*α*T is overwhelmingly the explanation for elevated BST. Raising additional questions, their cohort was strikingly female-predominant, showed an unexpected association with thyroid disease, and reported (predominantly low-grade) anaphylaxis in over half of the patients. These observations frame two recurring themes in the collection: the need to interpret BST against genotype and the extent to which the trait truly confers excess anaphylaxis risk.

Two complementary reviews addressed the diagnostic problem directly. Crupi et al*.* argued for a context-dependent reading of BST levels, noting that H*α*T adds approximately 9–10 ng/mL per supernumerary *α*-allele, such that thresholds of >20 and >200 ng/mL lose meaning without genotype. McMurray et al. expanded on this by providing evidence that elevated BST, interpreted against an individual's tryptase genotype, is the single most accurate screening test for SM and that this is required to apply the established BST thresholds. The authors highlighted an easily overlooked management consequence: because tyrosine kinase inhibitors do not eliminate wild-type tryptase production, patients with both SM and H*α*T are not expected to normalize their BST levels with disease-modifying therapy, which puts them at risk of inappropriate dose escalation or premature treatment discontinuation if their genotype is unknown.

The relationship between H*α*T and MCDs was dissected most explicitly by Chantran and Arock and by Polivka et al*.* Both research groups confirmed a consistent overrepresentation of H*α*T across MCD cohorts, accompanied by higher rates of anaphylaxis and a shift toward low-burden, noncutaneous subtypes. Chantran and Arock provided three explanations—H*α*T as a BST modifier, a symptom modifier, and a candidate causal factor—and suggested that much of the apparent enrichment reflects ascertainment bias rather than a true pathophysiological association. Polivka et al*.* translated this uncertainty into practical guidance: they suggested performing first-line peripheral blood H*α*T genotyping and *KIT* D816 V testing. Due to the low sensitivity of peripheral blood *KIT* assays in cases without skin involvement, they noted that negative results cannot exclude disease. Given the low estimated clonal disease prevalence in carriers, they argued against routine family screening.

Beyond the anaphylaxis and MCD settings, Lang reviewed H*α*T in food allergy, concluding that although food allergy prevalence in H*α*T appears to be comparable to that in the general population, *α*-tryptase behaves as a gene-dose-dependent risk factor for reaction severity (paralleling its effect in Hymenoptera venom anaphylaxis). As such, *TPSAB1* genotyping represents a candidate biomarker for reaction severity.

Vainer and Peterson made the case that GI symptoms of H*α*T (which mimic irritable bowel syndrome) likely represent a distinct, PAR-2-driven entity. The authors offered a practical mediator-directed treatment ladder while flagging emerging targeted therapies. This was given substance by Galeas-Pena et al., who demonstrated that H*α*T-associated IBD is marked by increased intestinal MC abundance and selective upregulation of MRGPRX2 and SIGLEC-8, suggesting an MC-centric, IgE-independent endotype. Finally, Simeone et al. presented a unifying model of H*α*T in which *α*/*β*-tryptase heterotetramers acting through EMR2 and PAR-2 modify mucosal immunity. The authors also highlighted the field's limitations: the unresolved question of causality, incomplete penetrance, and the near-total absence of data from individuals of non-European ancestry.

While we have learned much about H*α*T over the past decade, as with any new discovery, continued investigation is required. A number of salient observations have been highlighted in this series; however, additional important questions have also been raised. The preponderance of evidence supports our current understanding of H*α*T as a common modifier of mast cell-mediated immediate hypersensitivity, which provides a unifying explanation of the multiple associations reported between H*α*T and clonal mast cell disease, along with non-clonal mast cell-mediated phenotypes. Importantly, data support the concept that H*α*T is simply a naturally occurring overexpression model of alpha-tryptase and thus exists on the spectrum of alpha-tryptase expression in humans. The manner in which alpha-tryptase overexpression may contribute to mast cell-dependent pathology may occur via the unique characteristics of tryptase heterotetramers, the increased number of mast cells in tissues reported in symptomatic individuals with H*α*T, or some other, as yet uncharacterized, mechanism. Because human tryptases—and indeed even human mast cells, despite having ancient evolutionary roots—are quite recently evolved and unique to humans, respectively, we have much left to learn.
